# Post-Embryonic Induction of *ATML1-SRDX* Alters the Morphology of Seedlings

**DOI:** 10.1371/journal.pone.0079312

**Published:** 2013-10-25

**Authors:** Shinobu Takada

**Affiliations:** Department of Biological Sciences, Graduate School of Science, Osaka University, Toyonaka, Osaka, Japan; Universidad Miguel Hernández de Elche, Spain

## Abstract

*Arabidopsis thaliana MERISTEM* LAYER *1* (*ATML1*), an HD-ZIP class IV homeobox gene, is one of the key regulators promoting epidermal cell differentiation in *Arabidopsis thaliana*. We recently showed that *ATML1* was able to confer an ectopic shoot epidermis cell fate to non-epidermal tissues of seedlings, suggesting that *ATML1* is a master regulator of epidermal cell fate. To further assess the roles of *ATML1* and its homologs in epidermal cell differentiation, I generated transgenic plants expressing *ATML1* fused with a transcriptional repressor sequence (*ATML1-SRDX*). Estradiol-induced expression of *ATML1-SRDX* in the seedlings decreased transcript levels of several epidermis-related genes. Moreover, these transgenic plants exhibited phenotypes such as increased permeability to a hydrophilic dye and fusion of leaves and cotyledons, which are reminiscent of epidermis and/or cuticle-deficient mutants. Epidermal cell morphology was severely affected in the strong lines: filamentous protrusions were formed on the surface of the cotyledons. Marker gene analyses showed that these protrusions did not have epidermis, mesophyll, root hair, or trichome cell identity, suggesting that post-embryonic expression of *ATML1-SRDX* was sufficient to alter cell identity in pre-existing protodermal cells of the cotyledons. Taken together, these results suggest that *ATML1* and/or its target genes are not only necessary for the initial specification of epidermal cell fate but also may be necessary for the maintenance of epidermal cells in later stages.

## Introduction

Molecular genetic studies in plants and animals have revealed that cell-type-specific transcription factors play key roles in determining cell fates through regulation of gene expression. *ATML1* is one of the key transcriptional regulators that promote epidermal cell differentiation in *Arabidopsis thaliana* [1–3]. *ATML1* belongs to the HD-ZIP class IV homeodomain protein family, and its mRNA is detected in the outermost cell layer from the early stages of development [1,4,5]. Mutations in *ATML1* and its closest homolog *PROTODERMAL FACTOR2* (*PDF2*) induced the formation of leaves lacking an epidermis, suggesting that they are redundantly required for epidermal cell differentiation in shoots [2]. Moreover, our overexpression experiments showed that *ATML1* was able to confer an ectopic shoot epidermal cell fate to non-epidermal tissues of seedlings, suggesting that *ATML1* functions as a master regulator of epidermal cell differentiation [3]. Several *ATML1* homologs were also expressed in the epidermis, suggesting the possible involvement of these homologs in epidermal cell differentiation [6]. In particular, several ATML1 homologs and ATML1 can bind to a common binding site known as L1 box [6,7]. L1 box is an eight-base-pair sequence often found in the promoters of epidermis-specific genes [7]. Thus, ATML1 homologs may also positively regulate the expression of epidermis-specific genes via binding to the L1 box and thereby promote epidermal cell differentiation. However, the actual roles of *ATML1* homologs in epidermal cell differentiation remain unclear because the effects of multiple loss-of-function mutations in the *ATML1* homologs have yet to be examined.

I postulated that dominant repression of target genes for ATML1 would induce phenotypes that resemble those of multiple knockouts of the *ATML1* homologs if they shared similar binding sites. To further assess the roles of ATML1 and its homologs in post-embryonic development, I expressed ATML1 fused with the transcriptional repressor sequence SRDX using an estradiol-inducible gene expression system [8,9]. Dominant-negative repression of target genes using SRDX has been widely used to assess the roles of functionally redundant transcription factors [9–14].

The results showed that *ATML1-SRDX* decreased expression of epidermis-specific genes. Moreover, *ATML1-SRDX* expressing plants exhibited a range of phenotypes related to defects in epidermal cell differentiation, which were more severe than those observed in the *atml1-1;pdf2-1* double mutant [2]. In the strong *ATML1-SRDX* lines, the morphology of the seedlings was severely affected with the formation of unusual protrusions on the surface of the cotyledons. Therefore, post-embryonic downregulation of target genes for ATML1 appears to be sufficient to alter the cell identity of pre-existing protodermal/epidermal cells in the cotyledons, suggesting that *ATML1* and/or its target genes may be necessary not only for the initial specification of epidermal cell fate but also for the maintenance of epidermal cell fate in later stages.

## Materials and Methods

### Plant materials and growth conditions

*proATML1-nls-3xGFP* was previously described [5]. *proATML1-nls-3xGFP* in the Columbia background was used as the wild type, unless otherwise indicated. *STOMAGEN-GUS* was previously described [15] and was a gift from Prof. Tatsuo Kakimoto (Osaka University, Japan). *atml1-1;pdf2-1* was previously described [2] and was kindly provided by Prof. Taku Takahashi (Okayama University, Japan). *GL1-GUS* was previously described [16] and was provided by the Arabidopsis Biological Resource Center (Stock number: CS8850).

For the phenotypic and expression analyses of seedlings, plants were grown on Murashige and Skoog (MS) media with 1% sucrose and 0.4% phytagel (Sigma-Aldrich, St. Louis, USA) in constant light conditions under white fluorescent light at 23°C. Sown seeds were kept for 2 days at 4°C and then moved to 23°C, which was defined as day 0 after sowing. For estradiol treatment, plants were germinated and grown on MS-phytagel plates containing 10 μM β-estradiol unless otherwise indicated. β-estradiol was dissolved in dimethyl sulphoxide (DMSO) as a stock solution of 100 mM. The same volume of DMSO was added to MS media for control experiments. 

### Plasmid construction and transgenic plants

To obtain estradiol-inducible lines, the G10 promoter in the pER8 vector was replaced with a promoter region of *AtRPS5A* from −1571 to +113 relative to the transcription start site (proRPS5A/pER8) [8,17]. Two oligonucleotides, 5′-CTACGTATCTGCTGCTCTTGATCTTGATCTTGAGCTTAGACTTGGATTCGCTTAAA-3′ and 5′-CTAGTTTAAGCGAATCCAAGTCTAAGCTCAAGATCAAGATCAAGAGCAGCAGATACGTAGGGCC-3′, were annealed and inserted into the ApaI and SpeI sites of proRPS5A/pER8 (proRPS5A-SRDX/pER8). The *ATML1* coding sequence lacking a stop codon was amplified using primers 5′-TTTCTCGAGAAAATGTATCATCCAAACATGTTC-3′ and 5′-TTTGGCGCGCCCGGCTCCGTCGCAGGCCAGAG-3′, and inserted into the XhoI and AscI sites of proRPS5A-SRDX/pER8, in frame with the SRDX sequence (proRPS5A-ATML1-SRDX/pER8).

proRPS5A-ATML1-SRDX/pER8 was used to transform the *proATML1-nls-3xGFP* line. A total of 14 of the 22 *proRPS5A-ATML1-SRDX/pER8* lines exhibited abnormal phenotypes when grown in the presence of 10 μM estradiol. Nine of the 14 lines showed fusion of cotyledons and leaves, and 13 of the 14 lines formed unusual protrusions on the surface of the seedlings. Three independent lines showing representative phenotypes were selected, and homozygous lines were established in the T3 generation. These transgenic plants did not show an abnormal phenotype in the absence of estradiol and in the presence of DMSO, and 10 μM estradiol did not affect the growth of wild-type and *proATML1-nls-3xGFP* plants (data not shown). The *ATML1-SRDX* seeds are available upon request.

For the marker line analyses, the homozygous *proRPS5A-ATML1-SRDX/pER8* lines 1 and 3 were crossed with *STOMAGEN-GUS* and *GL1-GUS*, respectively. F2 plants were used for *GUS* expression analysis. To generate the *EXP7-GUS* marker (described by Cho and Cosgrove, 2002) [18], a 1463-bp sequence ranging from −1423 to +40 relative to the transcription start site of *AtEXP7* was amplified using PCR with primers 5′-TTTTAAGCTTGTTTGTTCGAAATCACAAACTCTCAATTTC-3′ and 5′-TTTTCTAGATTCTAGCCTCTTTTTCTTTATTCTTAGGGTTTG-3′, and inserted into the HindIII and XbaI sites of the pBI101 vector (Clontech, Mountain View, USA). The resulting construct was used to transform *proRPS5A-ATML1-SRDX/pER8* line 3, and T2 plants were used for GUS expression analysis.

### GFP expression analysis

GFP fluorescence was observed using a confocal laser scanning microscope Fluoview FV300 (Olympus, Tokyo, Japan), with excitation at 488 nm and collection at 510–530 nm.

### Histological analysis

For GUS staining, the samples were soaked in 90% acetone for 20 min on ice, rinsed twice with a washing solution (50 mM sodium phosphate buffer, pH 7.2; 0.5 mM potassium ferrocyanide; 0.5 mM potassium ferricyanide; and 10 mM EDTA) and incubated in a staining solution (washing solution with 0.5 mM X-Gluc) at room temperature or 37°C for 20 h. Samples were fixed overnight at 4°C in 1% glutaraldehyde and 3% formaldehyde in phosphate-buffered saline (pH 7.0) and dehydrated through an ethanol series. For whole-mount observation, the samples were mounted with a clearing solution (8 g chloral hydrate, 1 ml glycerol, and 2 ml water), and photographed.

To create histological sections, the samples were embedded in Technovit 7100 (Heraeus Kulzer, Wehrheim, Germany) and cut to a thickness of 4–6 μm with a RM 2155 rotary microtome (Leica Microsystems, Wetzlar, Germany) equipped with a S35 disposable blade (Feather, Osaka, Japan). The sections were stained with 0.1% toluidine blue, coverslipped with Eukitt mounting medium (O. Kindler, Freiburg, Germany), and photographed.

The nuclei of the cotyledons were stained with 1 × SYBR Gold nucleic acid gel stain (Molecular Probes, Eugene, USA) in 4% formaldehyde and 5% glycerol in phosphate-buffered saline (pH 7.0), and observed using a confocal laser scanning microscope LSM 710 (Carl Zeiss, Oberkochen, Germany), with excitation at 488 nm and collection at 533–544 nm.

For the wounding treatment, the distal halves of the cotyledons of seven-day-old wild-type (Columbia) seedlings were cut off with scissors. Fifteen of 33 wounded cotyledons produced calli after 7 days.

The toluidine blue test was performed as previously described [19].

Primary root length was measured using the ImageJ 1.43 software (National Institutes of Health, USA).

### Quantitative reverse-transcriptase polymerase chain reaction (RT-PCR) analyses

For the quantitative RT-PCR analyses, total RNA was extracted from seven-day-old seedlings using the RNeasy Plant Mini Kit (Qiagen, Venlo, Netherlands). For RNA extraction, 10–30 plants were used for each sample. Total RNA (0.4–0.8 μg) was treated with one unit of Amplification grade DNase I (Invitrogen, Carlsbad, USA) and reverse-transcribed using Superscript II reverse transcriptase (Invitrogen) with oligo-dT primers in a volume of 20 μl according to the manufacturer’s instructions. Real-time PCR reactions were performed using FastStart Universal SYBR Green Master (Roche, Basel, Switzerland) on an ABI 7300 Real-Time PCR System (Applied Biosystems, Foster City, USA). Two microliters of a 32-fold dilution of the cDNA reaction was used for PCR in a volume of 20 μl. Three biological replicates were used, and each sample was analyzed in duplicate. Data analyses were performed using the Sequence Detection Software version 1.3.1 (Applied Biosystems). Absolute quantification of gene expression was performed using a dilution series of cloned cDNA. Analysis of the melting curves was performed for each PCR reaction to ensure amplification of a single product. No significant signal was detected in the minus reverse transcriptase control (data not shown). The *PP2A* gene, which is a stably expressed gene, was used as a reference to normalize the expression levels [20]. Primers used for amplification were as follows: 5′-ACTGTCGAGCGGATTAAAGC-3′ and 5′-ATCAAGATCAAGAGCAGCAGATAC-3' for *ATML1-SRDX*; 5′-TTAAAGCCGCTCTGGCCTGCGAC-3′ and 5′-AGGTGCGTTCTTGACTTCCTTTGGAG-3′ (located in the 3′-untranslated region) for endogenous *ATML1*; 5′-TCCGCGAAGAGATTGATAGG-3′ and 5′-AGATCAAGCGAACGAGAAGG-3′ for *PDF2*; 5′-TTTTGTGCAGGAGTGAGTGC-3′ and 5′-TCCCTCCCGTTAGCAATATG-3′ for *HDG2*; 5′-TTCCGCCACCGCAAAAACCAATG-3′ and 5′-TGCCGCGTGGAAGCAAAAATGC-3′ for *FDH*; 5′-TGAGTTTTGCCGTTTGGGCTCTC-3′ and 5′-TGTGGAGTTGGCGTGTGTGATGG-3′ for *PDF1*; 5′-AGGAATATCGCTCGAGATGG-3′ and 5′-TGTCTCCCGAATCCTTTGAG-3′ for *CER5*; 5′-GCAACAGGGCAAAAACCGCTTC-3′ and 5′-TCAAGCGATGCACAAGCCTTTCG-3′ for *ZLL*; 5′-TCGCGCCAAGATTGAAGCTGGAAAG-3′ and 5′-AAACGAAAACCGCGAGCTCAATGC-3′ for *CRE1*; and 5′-TAACGTGGCCAAAATGATGC-3′ and 5′-GTTCTCCACAACCGCTTGGT-3′ for *PP2A*.

## Results and Discussion

### Post-embryonic induction of *ATML1-SRDX* caused abnormal phenotypes related to defects in epidermal differentiation

**Figure 1:**
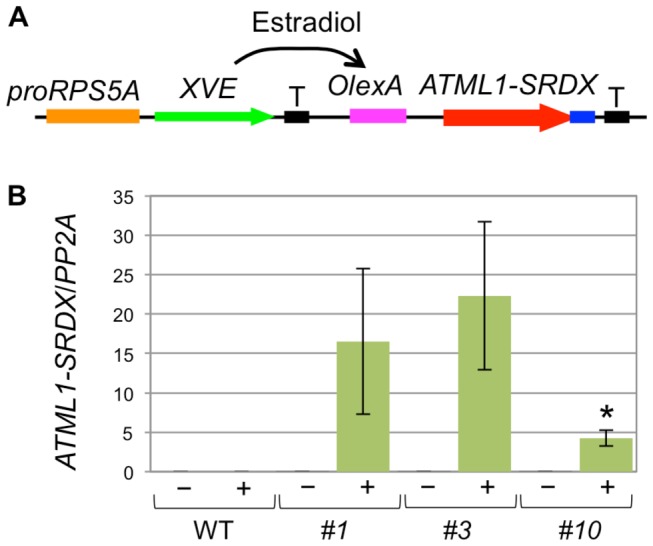
Estradiol-induced expression of *ATML1-SRDX.* (A) Representation of an estradiol-inducible gene expression system used to express ATML1-SRDX. XVE (LexA DNA-binding domain/VP16 activation domain/estrogen receptor) was expressed under the control of the RPS5A promoter (proRPS5A) [8,17]. After treatment with estradiol, XVE binds to the LexA binding site (OlexA) and activates ATML1-SRDX expression. For simplicity, hpt (conferring hygromycin resistance) was omitted from this figure. T, transcriptional terminator. (B) ATML1-SRDX expression in seven-day-old seedlings of estradiol-inducible lines (#1, #3, and #10) and the wild type (WT) grown in the absence (−) or presence (+) of 10 μM estradiol. Data were normalized to the amount of PP2A [20]. Values are the mean ± SEM from three biological replicates. Asterisks indicate a statistically significant difference relative to the wild type (unpaired two-tailed t-test; p < 0.05). Note that transcripts of ATML1-SRDX were not detected in the wild type and were almost absent in lines 1, 3, and 10 without estradiol treatment.

To understand the roles of *ATML1* and its homologs in epidermal cell differentiation in the seedlings, I generated transgenic plants carrying the estradiol-inducible *ATML1* gene fused to the *SRDX* repressor sequence, which can convert a transcriptional activator into a repressor (ATML1-SRDX; Figure 1A) [8,9]. I established three independent lines showing relatively stronger (#1, #3) and weaker (#10) expression of *ATML1-SRDX* after estradiol treatment (Figure 1B).

**Figure 2:**
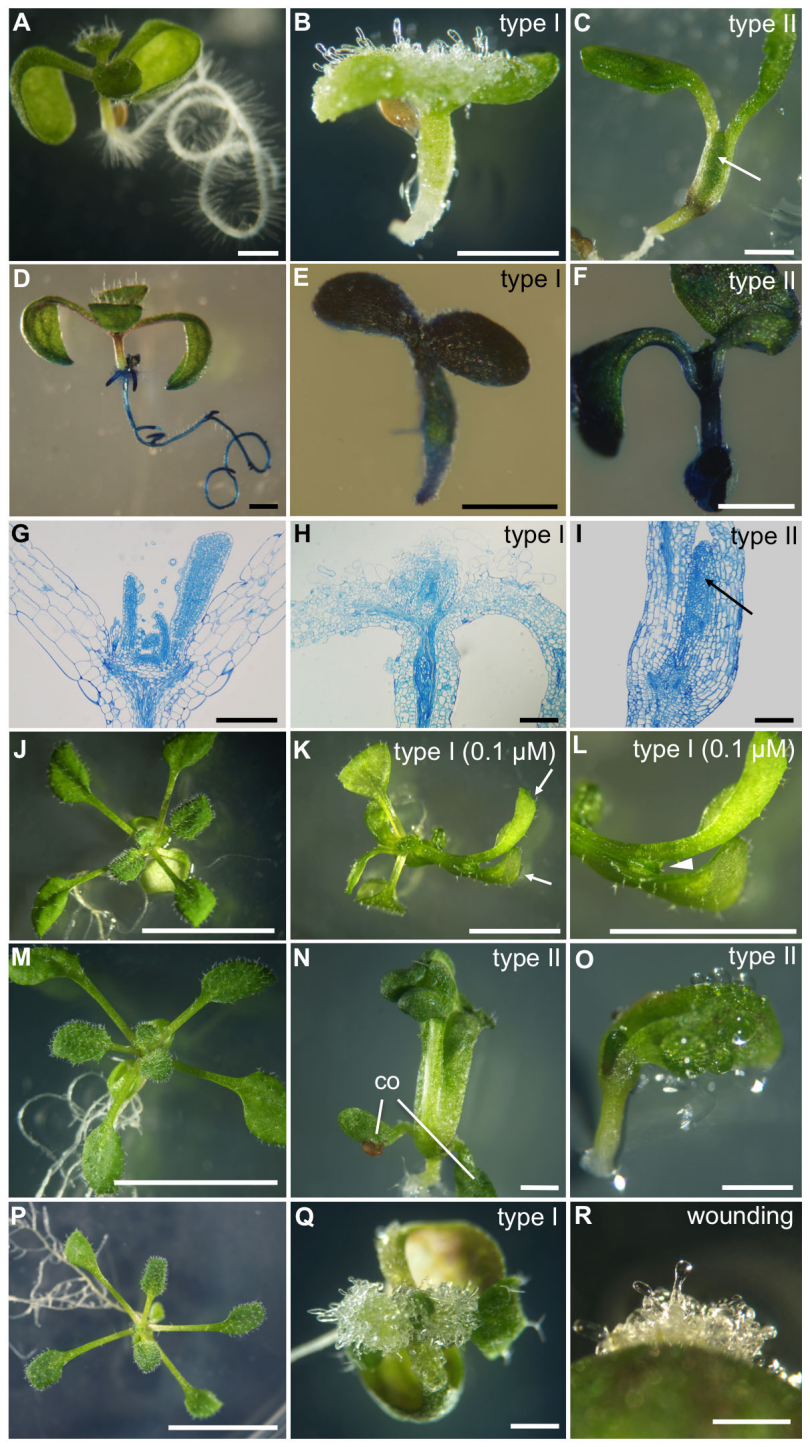
Phenotypes of *ATML1-SRDX* lines. (**A**–**C**) Seven-day-old seedlings of the wild type (A), a type I *ATML1-SRDX* line (B), and a type II *ATML1-SRDX* line (C) grown on MS-phytagel plates containing 10 μM estradiol. The arrow indicates fused leaves formed at the shoot apex. (**D**–**F**) Seven-day-old seedlings of the wild type (D), a type I *ATML1-SRDX* line (E), and a type II *ATML1-SRDX* line (F) grown with 10 μM estradiol and stained with toluidine blue (blue color). (**G**–**I**) Longitudinal sections of seven-day-old wild-type (G), type I *ATML1-SRDX* (H), and type II *ATML1-SRDX* seedlings (I) grown with 10 μM estradiol and stained with toluidine blue. The arrow in I indicates a leaf primordium. In Figure 2G–2I, toluidine blue staining was performed after sectioning to visualize the cell boundaries. (**J**) A 16-day-old seedling of the wild type grown with 0.1 μM estradiol. (**K**) Fused leaf development (arrows) in a type I *ATML1-SRDX* line grown with 0.1 μM estradiol for 16 days. (**L**) Magnified view of the fused leaves in K. The arrowhead indicates a junction of the fused leaves. (**M**) A 14-day-old seedling of the wild type grown with 10 μM estradiol. (**N**) Fused leaf formation in a type II *ATML1-SRDX* line grown with 10 μM estradiol for 14 days. co, cotyledon. (**O**) An eight-day-old seedling of a type II *ATML1-SRDX* line grown with 10 μM estradiol. Droplets of water were seen on the surface of the seedling. (**P**, **Q**) 14-day-old seedlings of the wild type (P) and a type I *ATML1-SRDX* line (Q) germinated on estradiol-free MS plates and transferred to 10 μM estradiol-containing plates after 5 days. Filamentous protrusions were formed on the surface of the leaves. (**R**) Calli formation from the cut end of a wild-type Columbia cotyledon. Scale bars, 1 mm for A–F, N, O, Q, R; 1 cm for J, M, P; 5 mm for K, L; and 200 μm for G–I.

In the strong lines (defined as type I), root growth was arrested and filamentous transparent protrusions, which resembled callus-like tissues often observed at the cut end of wild-type cotyledons, were formed on the surface of the cotyledons [Figure 2B and 2R; 96.8% of the seedlings in #1 (n = 63) and 83.8% of the seedlings in #3 (n = 68)]. Histological analysis showed that the cell arrangement was disorganized (Figures 2H and S1). Treatment of the strong lines with a lower concentration of estradiol (0.1 μM) often resulted in plants showing fusion of the leaves [Figure 2K and 2L; 29.0% of the seedlings in #1 (n = 31) and 61.5% of the seedlings in #3 (n = 26)], as seen in cuticle-deficient mutants [21–25]. To determine the effect of *ATML1-SRDX* expression on leaf development, type I plants were germinated on estradiol-free MS-phytagel plates and transferred to 10 μM estradiol-containing plates. In these plants, filamentous cells were formed on the surface of the leaves [Figure 2Q; 56.7% of the seedlings in #1 (n = 30) and 69.0% of the seedlings in #3 (n = 29)] and the cotyledons [53.3% of the seedlings in #1 (n = 30) and 65.5% of the seedlings in #3 (n = 29)], indicating that formation of these filamentous cells by *ATML1-SRDX* expression was not confined to the cotyledons.

Treatment with 10 μM estradiol had less effect on root growth in the weak lines (type II) compared with the strong lines (Figure 2C). Leaf development was variably affected in these plants and ranged from arrested in leaf development [11.1% of the seedlings in #10 (n = 72)] to the development of fused leaves [68.1% of the seedlings in #10 (n = 72)] (Figure 2C, 2I, 2N, and 2O). In some extreme cases, the leaves were fused with the petiole of the cotyledons (Figure 2C and 2I).

Permeability to the hydrophilic dye toluidine blue was increased in the cotyledons of the three *ATML1-SRDX* lines [Figure 2E and 2F; 100% of the seedlings showed staining in #1 (n = 24) and #3 (n = 51) and 85.6% of the cotyledons in #10 (n = 118)], whereas little toluidine blue staining was observed on the cotyledons of wild-type plants [Figure 2D; 3.9% of the cotyledons showed staining (n = 128)], suggesting a defect in the cuticular barrier in the *ATML1-SRDX* induced plants. Droplets of water were often seen on the surface of the *ATML1-SRDX* line 10 seedlings [Figure 2O; 33.3% of the seedlings in #10 (n = 72)], suggesting that water can be exuded from the surface in the absence of a proper epidermis or cuticle.

In summary, *ATML1-SRDX* caused abnormal phenotypes including increased permeability of the epidermis and fusion of the cotyledons/leaves. These findings suggest that post-embryonic expression of *ATML1-SRDX* was sufficient to mimic epidermis-deficient mutant phenotypes.

**Figure 3:**
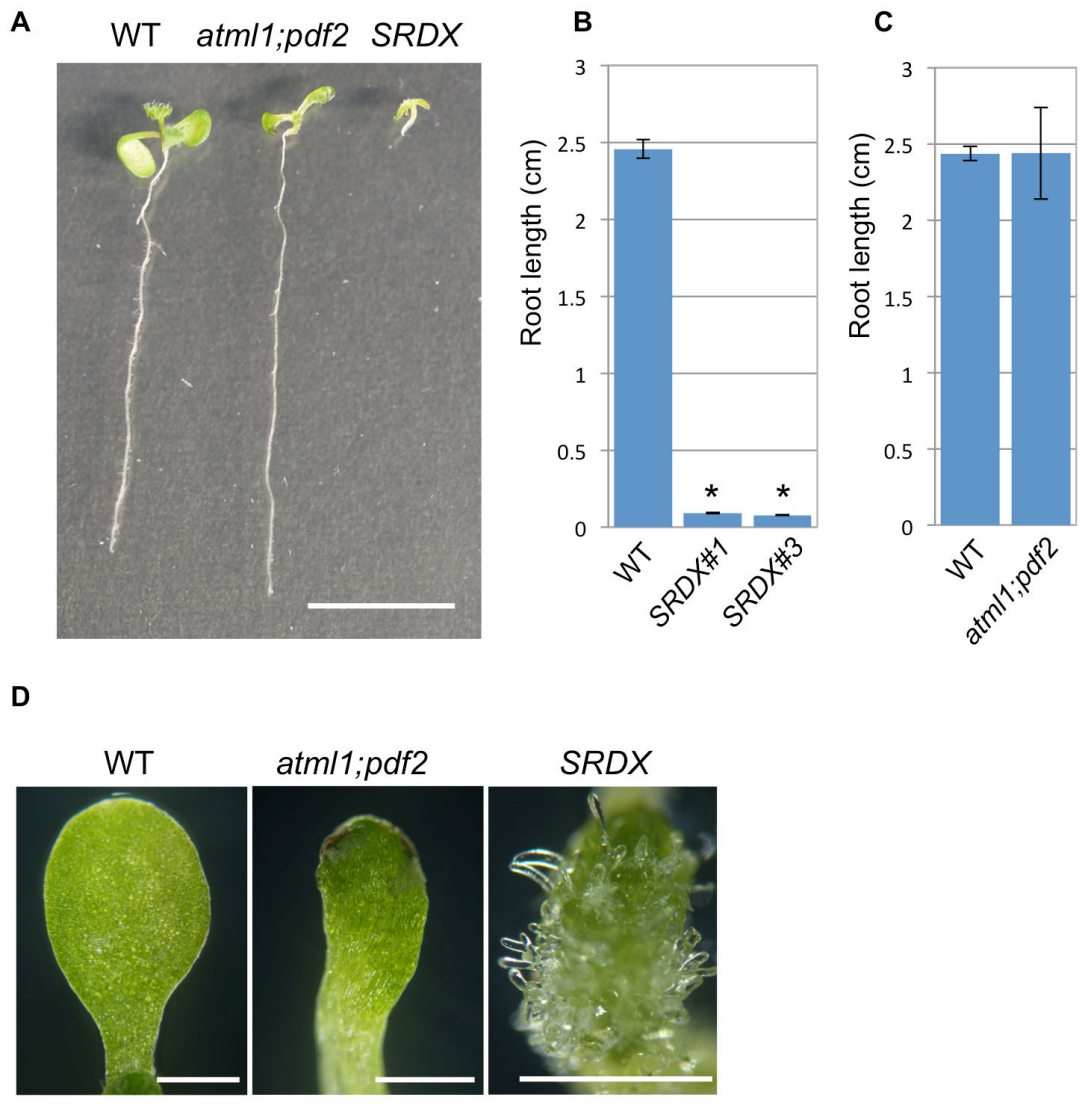
Phenotypes of strong *ATML1-SRDX* lines compared with those of *atml1-1*;*pdf2-1*. (**A**) Seven-day-old seedlings of the wild-type Columbia (WT), *atml1-1;pdf2-1* (atml1;pdf2), and a type I *ATML1-SRDX* line (SRDX) grown with 10 μM estradiol. (**B**, **C**) Quantification of the root length of seven-day-old type I *ATML1-SRDX* line (*SRDX#1* and *SRDX#3*; B) and *atml1-1;pdf2-1* (*atml1;pdf2*; C) seedlings grown with 10 μM estradiol. Values are the means ± SEM (n = 7–30). Asterisks indicate a statistically significant difference relative to the wild type grown under the same conditions (unpaired two-tailed *t*-test; p < 0.05). Columbia was used as the wild type in C. (**D**) Comparison of the cotyledon morphology of the wild type (WT), *atml1-1;pdf2-1* (atml1;pdf2), and a type I *ATML1-SRDX* line (SRDX) grown with 10 μM estradiol for 7–8 days. Scale bars, 1 cm for A and 1 mm for D.

The phenotypes of the strong *ATML1-SRDX* lines were more severe than those of *atml1-1;pdf2-1* in terms of root growth arrest and the formation of filamentous protrusions on the cotyledons. The primary root length of *ATML1-SRDX* lines 1 and 3 was significantly shorter than that of the wild type, whereas *atml1-1;pdf2-1* did not exhibit a short root phenotype (Figure 3A–3C). Cotyledons of *atml1-1;pdf2-1* seedlings did not form the filamentous protrusions that were observed in the strong *ATML1-SRDX* lines [Figure 3D; 0% of the seedlings observed (n = 20)]. These observations may suggest a possible redundancy among HD-ZIP class IV transcriptional regulators, considering that some HD-ZIP class IV genes are also expressed in the roots [6]. Instead, it is also possible that these severe seedling phenotypes may represent those of the strong mutant alleles of *atml1;pdf2*, as one cannot exclude the possibility that some remaining activity of ATML1 and PDF2 is present in *atml1-1*;*pdf2-1*.

### Transcripts of several epidermis-specific genes were reduced in *ATML1-SRDX*-expressing plants

Overexpression of *ATML1* promotes the expression of several epidermis-specific genes [3]. In order to determine whether or not *ATML1-SRDX* decreased the expression of epidermis genes, I examined the expression of representative L1 box-containing genes that were uniformly expressed in the shoot epidermis.

**Figure 4:**
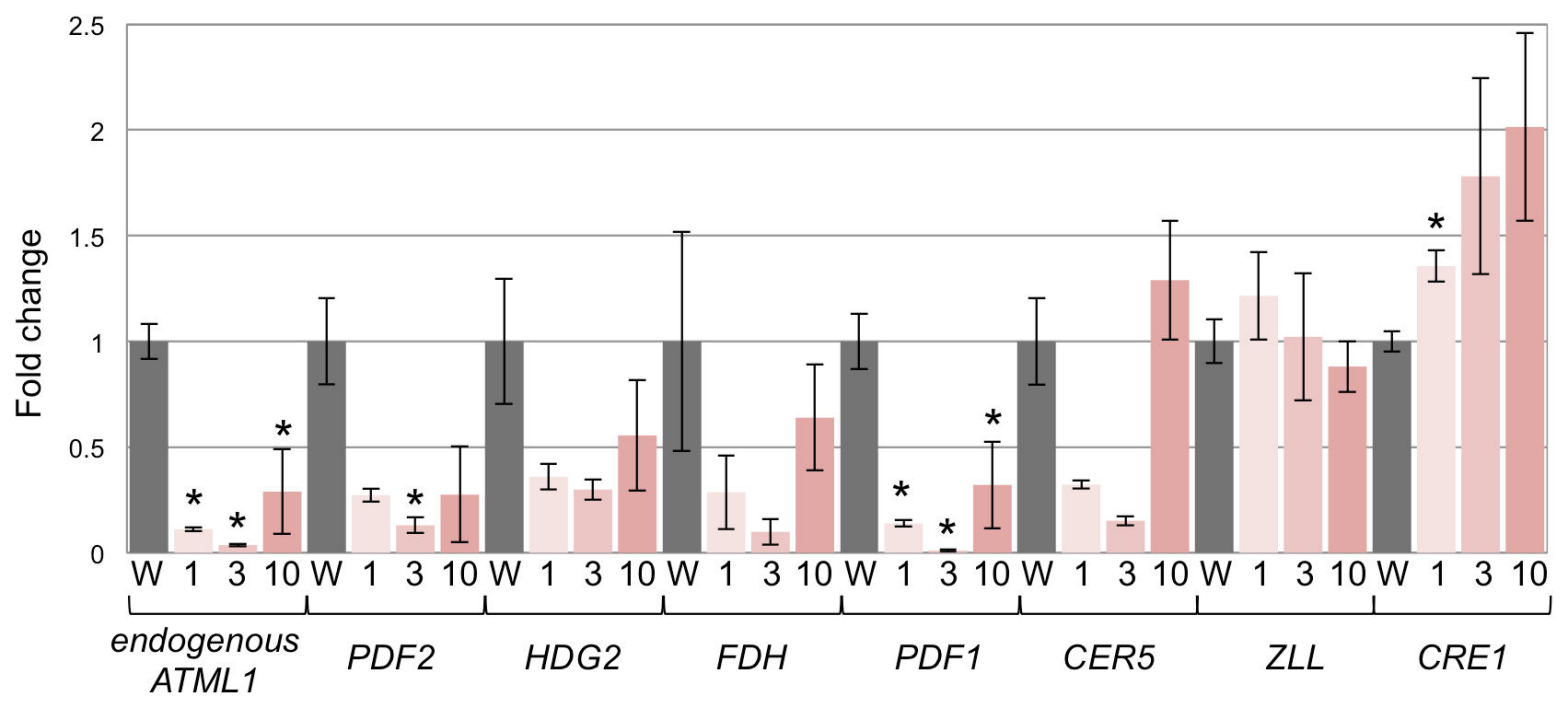
Quantitative RT-PCR analyses in *ATML1-SRDX* lines. Real-time RT-PCR analysis of the expression of endogenous ATML1, PDF2, HDG2, FDH, PDF1, CER5, ZLL, and CRE1 in seven-day-old seedlings of estradiol-inducible lines 1, 3, and 10 (1, 3, and 10, respectively) and the wild type (W) grown in the presence of 10 μM estradiol. Expression levels were normalized to PP2A expression [20], and expression in the wild type was set to 1. Values are the mean ± SEM from three biological replicates. Asterisks indicate a statistically significant difference relative to the wild type (unpaired two-tailed t-test; p < 0.05). CRE1 mRNA expression was significantly increased in ATML1-SRDX line 1.

Quantitative RT-PCR analyses indicated that expression of the endogenous *ATML1* and *PROTODERMAL FACTOR1* (*PDF1*) genes was significantly decreased in all the *ATML1-SRDX*-expressing lines (Figure 4) [26]. In addition, expression of the *PDF2* gene was significantly decreased in *ATML1-SRDX* line 3 (Figure 4). Expression levels of the *ECERIFERUM 5* (*CER5*), *FIDDLEHEAD* (*FDH*), and *HOMEODOMAIN GLABROUS2* (*HDG2*) genes were low in the *ATML1-SRDX* lines compared with the wild type in three independent experiments, but the difference was not statistically significant (Figure 4) [6,27–29]. In contrast, expression of the central domain-specific *ZWILLE* (*ZLL*) and *CYTOKININ* RESPONSE *1* (*CRE1*) genes was not significantly decreased in the *ATML1-SRDX* lines (Figure 4) [30–32].

These results show that *ATML1-SRDX* was able to decrease the expression of several epidermis-related genes, and further support the idea that *ATML1* functions, either directly or indirectly, as a positive transcriptional regulator of these L1 box-containing genes.

### Cell identity of the filamentous protrusions is unclear

**Figure 5:**
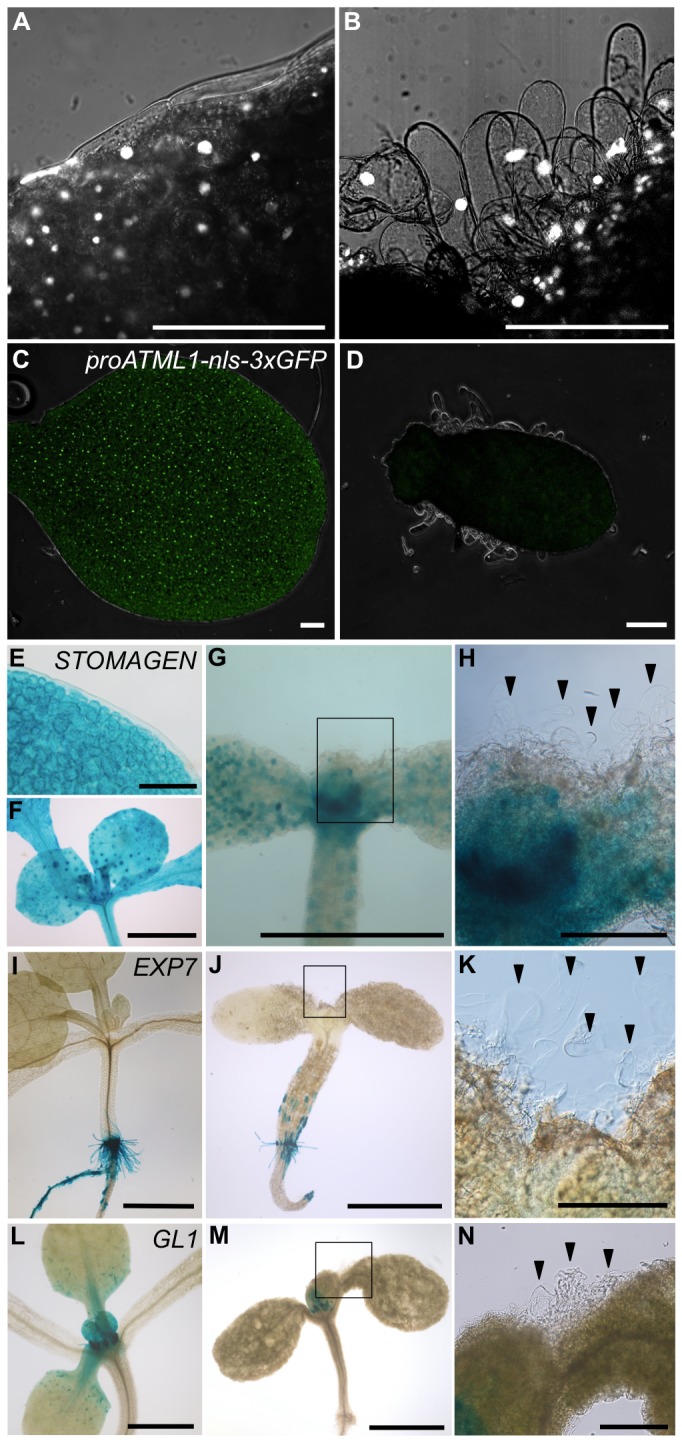
Marker gene analyses of the filamentous protrusions formed on the surface of *ATML1-SRDX* seedlings. (**A**, **B**) Confocal images of cotyledons from eight-day-old seedlings of the wild type (A) and *ATML1-SRDX* line 1 (B) grown with 10 μM estradiol. Nuclei were stained with SYBR Gold nucleic acid gel stain (white signals). (**C**, **D**) *proATML1-nls-3xGFP* expression (green nuclear signals) in cotyledons from seven-day-old seedlings of the wild type (C) and *ATML1-SRDX* line 1 (D) grown with 10 μM estradiol, observed under a confocal laser scanning microscope. Images were taken under the same confocal microscope settings. (**E**, **F**) Wild-type expression patterns of *STOMAGEN-GUS* (shown in blue) in a cotyledon (E) and the shoot apex (F). (**G**) *STOMAGEN-GUS* expression (shown in blue) in a seven-day-old seedling of the *ATML1-SRDX* line 1 grown with 10 μM estradiol. (**H**) Magnified view of the inset in G. (**I**, **J**) *EXP7-GUS* expression (shown in blue) in seven-day-old seedlings of the *ATML1-SRDX* line 3 grown with DMSO (I) and 10 μM estradiol (J). (**K**) Magnified view of the inset in J. (**L**, **M**) *GL1-GUS* expression (shown in blue) in seven-day-old seedlings of the wild type (L) and *ATML1-SRDX* line 3 (M) grown with 10 μM estradiol. (**N**) Magnified view of the inset in M. The arrowheads indicate filamentous protrusions. Scale bars, 100 μm for A, E; 200 μm for B–D, H, K, N; and 1 mm for F, G, I, J, L, M.

The protrusions formed in the strong lines consisted of large, transparent cells some of which showed clear nuclear staining (Figures 2B and 5B). Similar protrusions were also observed in the seedlings of the *desperado/wbc11* mutant, which was defective in the transport of cutin and wax monomers to the extracellular matrix, suggesting that these unusual protrusions were related to a defect in epidermal cell integrity [24].

In order to determine the identity of these protrusions, I analyzed marker gene expression. Expression of the epidermis marker *proATML1-nls-3xGFP* was severely reduced in the protrusions formed on the cotyledons, suggesting that these protrusions lost epidermal cell identity (Figure 5D) [5]. Moreover, these protrusions did not express the mesophyll marker *STOMAGEN-GUS*, the trichome marker *GL1-GUS*, or the root hair cell marker *EXP7-GUS* (Figure 5G, 5H; 5J, 5K; and 5M, 5N; respectively) [15,16,18]. Judging from the absence of marker gene expression, I speculate that these cells may represent a unique type of cells that have lost any specific cell identity.

## Conclusions

Post-embryonic induction of *ATML1-SRDX* caused a range of phenotypes resembling epidermis-deficient mutants. The seedling phenotypes in the strong lines were more severe than those of the *atml1-1;pdf2-1* double mutant seedlings [2], which may suggest a possible redundancy among HD-ZIP class IV transcriptional regulators.

Epidermal cells of the cotyledons are specified during embryogenesis [5]. However, post-embryonic expression of *ATML1-SRDX* was able to negatively influence epidermal cell differentiation of the cotyledons. Thus, this study raises the possibility that *ATML1* and/or its targets may be necessary not only for the initial specification of epidermal cell fate but also for the maintenance of epidermal cell fate in later stages.

## Supporting Information

Figure S1 **Effect of *ATML1-SRDX* on cell arrangement in the cotyledons.** Longitudinal sections of cotyledons from 7-day-old seedlings of the wild type (A) and *ATML1-SRDX* line 1 (B) grown with 10 μM estradiol and stained with toluidine blue. Scale bars, 100 μm.
(TIF)
