# Supplementary figures and images for: Post-Embryonic Induction of ATML1-SRDX Alters the Morphology of Seedlings
Source: PLoS One. 2013 Oct 25;8(10):e79312. doi: 10.1371/journal.pone.0079312 (PMC3808298; doi:10.1371/journal.pone.0079312)

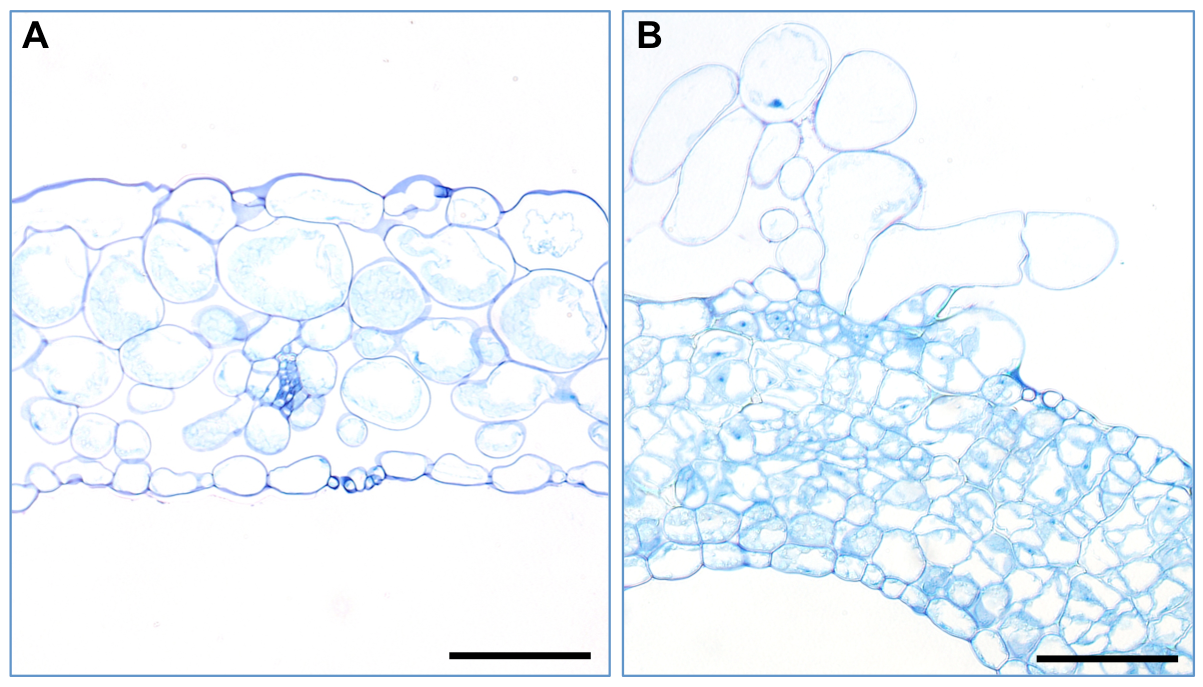

Supplement: Figure S1 — Effect of ATML1-SRDX on cell arrangement in the cotyledons. Longitudinal sections of cotyledons from 7-day-old seedlings of the wild type (A) and ATML1-SRDX line 1 (B) grown with 10 μM estradiol and stained with toluidine blue. Scale bars, 100 μm. (TIF) [file pone.0079312.s001.tif]
